# Epigallocatechin-3-gallate (EGCG) for Clinical Trials: More Pitfalls than Promises?

**DOI:** 10.3390/ijms12095592

**Published:** 2011-08-31

**Authors:** Derliz Mereles, Werner Hunstein

**Affiliations:** 1 Department of Cardiology, Angiology and Pneumology, University of Heidelberg, D-69120 Heidelberg, Germany; 2 Faculty of Medicine, University of Heidelberg, D-69120, Heidelberg, Germany; E-Mail: werner.hunstein@urz.uni-heidelberg.de

**Keywords:** epigallocatechin-3-gallate, green tea, bioavailability

## Abstract

Epigallocatechin-3-gallate (EGCG), the main and most significant polyphenol in green tea, has shown numerous health promoting effects acting through different pathways, as antioxidant, anti-inflammatory and anti-atherogenic agent, showing gene expression activity, functioning through growth factor-mediated pathways, the mitogen-activated protein kinase-dependent pathway, the ubiquitin/proteasome degradation pathway, as well as eliciting an amyloid protein remodeling activity. However, epidemiological inferences are sometimes conflicting and *in vitro* and *in vivo* studies may seem discrepant. Current knowledge on how to enhance bioavailability could be the answer to some of these issues. Furthermore, dose levels, administration frequency and potential side effects remain to be examined.

## 1. Introduction

Since our letter to the editor of *blood* in 2007 “Epigallocatechin-3-gallate (EGCG) in AL amyloidosis: a new therapeutic option?” [1], we have been confronted with followers and inveterate foes. Followers were the patients that followed the idea of drinking green tea as adjuvant to the current offered therapy options. Inveterate foes were the college physicians, especially all those that worked on scientific projects concerning this rare disease with poor prognosis and limited therapeutic alternatives. Patients took the decision to go on and try this over-the-counter choice, sometimes even hidden from the negative posture of their attending physicians. This made it possible to extend our analysis from a case report to a report of cases in a cohort of these patients [2]. Furthermore, based on these findings, as well as on precedent basic research [3–5] and in spite of the difficulties concerning ethical matters related to the discussion as to whether EGCG is a drug or just a dietary supplement, a world leading research group is planning to start the first clinical trial with EGCG in patients with AL amyloidosis [6,7].

**Figure 1:**
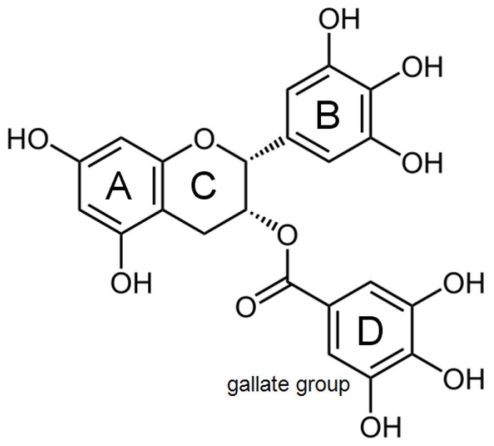
Chemical structure of EGCG. Some currently documented structure-function relationships are depicted in this figure. The pyrogallol-type structure on the B-ring induces apoptosis and possesses strong antioxidative activity undergoing autooxidation to form reactive oxygen species [10,11]. The galloyl moiety (D-ring, gallate group) is the critical structure in the inhibition of fatty-acid synthase leading to cytotoxicity in human cancer cells [12]. Both components B and D contribute to the exertion of biological activities related to the cell-surface 67 kDa laminin receptor [13,14]. EGCG molecular docking fractions for amyloid remodeling activity are still not known.

A quick look at EGCG and/or green tea on PubMed.gov [8] today results in a listing of more than 4000 publications since 1940. The first publications on EGCG alone as main polyphenol in green tea is dated 1985; an actualized review on health promoting effects of EGCG from green tea is currently available [9] (Figure 1). From these publications, the impression arises that EGCG could prevent, or even cure almost every disease: a panacea-related discredit cannot be denied. However, basic research provides clear support for hypotheses on the use of EGCG, and in spite of this fact, an apparent discrepancy between those results and the occurrence of awaited effects in clinical trials seems to linger on.

Thus, the aim of this review is to discuss known issues that could lead to failure in the clinical use of EGCG, starting with the concept of responders and non-responders, going through its erratic bioavailability and finally commenting on its possible side effects.

## 2. Responders *vs.* Non-Responders

Clinically relevant cytotoxic levels of EGCG in plasma [15] may not be reached *in vivo* through oral ingestion of green tea, green tea extracts or pure EGCG; health promoting targets of this polyphenol will then take place through other pathways. The decrease of β-pleaded protein deposits in cardiac amyloidosis due to the protein remodeling activity of EGCG [3–5] may be closely monitored by assessment of left ventricular mass and function [1–4,6]. The degree of responsiveness to EGCG may depend on its plasma and tissue bioavailability. Thus, since the latter may not be clinically possible in all cases at the moment, at least close controls of plasma levels of EGCG should be attempted.

### 2.1. Monitoring Left Ventricular Wall Thickness and Function

The reduction of left ventricular wall thickness in at least 2 mm [16], as well as left ventricular mass and improvement in global left ventricular function are expected changes in patients responders to EGCG [2] in cardiac involvement with protein deposits due to immunoglobulin light chain amyloidosis (AL). Consequently, improvement functional class assessed with the New York Heart Association (NYHA) classification can be observed [2].

However, not all patients with this targeted therapy will show the same effect. Our observations lead us to infer that there may be different types of responders to EGCG, as defined with the time to end-point (TEP, the interval until a 2 mm decrease in left ventricular wall thickness) could be documented. From the data in Table 2 [2] there were fast-responders: *n* = 4, with a TEP ≤ 3 months, slow-responders: *n* = 2, with a TEP ≤ 6 months, and very-slow-responders: *n* = 3, with a TEP ≤ 12 months. One patient showed the expected reduction in wall thickness with a 16 months delay.

As in almost every known therapeutic approach in internal medicine, non-responders may also be found. We understand that the concept of non-responders to EGCG is a very dynamic one, since this unstable polyphenol shows a very variable bioavailability [17]. After correcting factors that lead to low levels in plasma, a non-responder patient may turn out to be a responder. Therefore, follow-up assessment of left ventricular wall thickness every 3 months may be necessary to monitor closely the degree of responsiveness of patients under EGCG medication in cardiac amyloidosis.

### 2.2. Assessment of Plasma Levels of EGCG

Quantification of plasma EGCG levels is necessary in order to monitor bioavailability in each patient at therapy start, but also during follow-up when changes in doses are made or new factors appear that may affect blood levels during the course of the disease. Non-responder patient could rapidly develop to a responder status after correcting factors that lead to low EGCG levels in plasma. Moreover, measurement of EGCG levels in plasma is fundamental to monitor this therapeutic approach, since the effects of EGCG take place in a plasma level dependent manner [6].

Blood samples should be collected in EDTA containing tubes. As at pH above 7 EGCG oxidizes and dimerizes easily, blood samples must be immediately transferred to an ice bath and the plasma should be separated from the cellular fraction by centrifugation at 4 °C. Plasma samples in an ascorbate EDTA solution should be stored at −80 °C until assessment using high-performance liquid chromatography with colorimetric array detector analysis [18].

## 3. Bioavailability of EGCG

The rather poor bioavailability of EGCG needs to be considered when we extrapolate results obtained *in vitro* to situations *in vivo*. There are known and still unknown reasons for erratic EGCG bioavailability. Pharmacokinetic parameters are useful for selecting the dose and dose frequency for intervention. Most of the ingested EGCG apparently does not get into the blood, since absorption takes place in the small gut and substantial quantities pass from the small to the large intestine where it undergoes further degradation by the action of local microbiota [17,19–21].

The peak plasma concentrations of EGCG are reached in 1–2 h in healthy subjects with one oral dose in the morning after an overnight fasting period. These levels diminish gradually to undetectable levels in 24 h. The elimination half-life of EGCG takes place at 3.4 ± 0.3 h [17].

### 3.1. Factors Influencing EGCG Bioavailability

**Figure 2:**
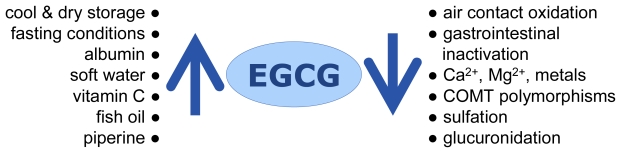
Factors influencing EGCG bioavailability: Factors enhancing plasma levels of EGCG are listed to the left, those that diminish bioavailability can be found on the right. Most of these factors are easily modifiable.

Considerable differences in the pharmacokinetic parameters in repeated experiments and among the individual subjects were observed in published information. The variations among the repeated experiments were rather large in some subjects [17]. The following factors may play a role (Figure 2).

#### 3.1.1. EGCG Oxidation Starts Already during Direct Contact with Air

EGCG is stable under anaerobic conditions *in vivo*, although the mechanisms for its stabilization have not yet been fully characterized. Catechins with a pyrogallol-type structure on the B-ring, such as EGCG, possess strong antioxidative activity and undergo autooxidation to form reactive oxygen species, resulting in polymerization and decomposition [11].

#### 3.1.2. EGCG May Undergo Gastrointestinal Inactivation

Several other factors, including temperature [22], oxygen concentration, antioxidant concentration, metal ions, among others also affect stability of EGCG [13]. Hard water with high concentrations of Ca^2+^ and Mg^2+^, or even drinking milk together with EGCG leads to its inactivation [23]. Furthermore, new findings on bioavailability have shown that EGCG absorption takes places mostly in the small intestine, and that in subjects with a functioning colon it passes to the large intestine where it is broken down to phenolic acids by the action of colonic microflora [19–21].

#### 3.1.3. EGCG Undergoes Liver Metabolism

After oral absorption, tea catechins undergo extensive methylation, glucuronidation, and sulfation. Rapid methylation of EGCG is catalyzed by liver cytosolic catechol-*O*-methyltransferase. Methylation decreases the hydrophilicity of catecholic compounds; further sulfation/glucuronidation of the methylated product is usually needed for the effective elimination of the methylation product from the body [24]. The level of methylation is influenced by polymorphisms of catechol-*O*-methyltransferase, but the scope of this effect has not yet been studied [17].

#### 3.1.4. EGCG Blood Transport and Stabilization Is Affected by the Level of Serum Albumin

Human serum albumin contributes to transport and stabilization by directly preventing EGCG oxidation through a reversible interaction with a higher affinity in alkaline conditions [11]. Hence, low serum albumin may decrease EGCG plasma levels.

### 3.2. Optimizing EGCG Bioavailability

**Figure 3:**
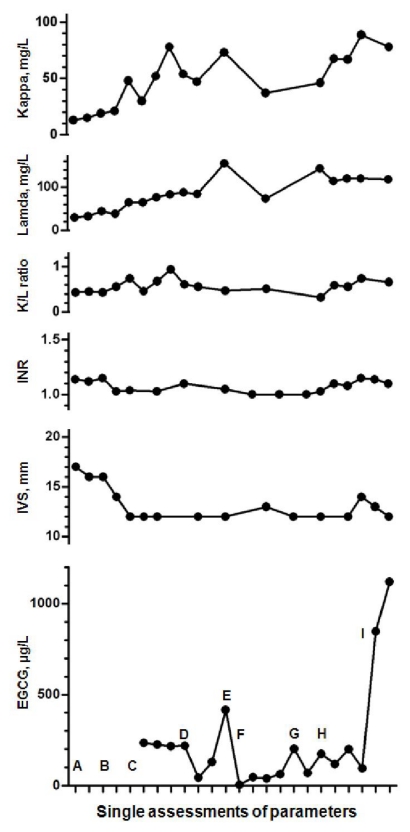
Single assessments of plasma levels of EGCG [6] available from 2008 on, measured 2 h after ingestion, depicted together with assessments of serum free light chains: Kappa, Lambda and K/L ratio, as well as international normalized ratio (INR) and interventricular septum thickness (IVS). EGCG assessments were undertaken 2 h after ingestion, referring to expected peak plasma levels [17,25]. (A) Therapy with oral melphalan plus high-dose dexamethasone until August 2006. No further conventional chemotherapy administered later on. (B) September 2006: start 1.5–2 L/day green tea (GT). (C) June 2008: Start 150 mg EGCG t.i.d. instead of GT, because of oral fluid intake restriction due to progressive renal failure. (D) October 2008: dosage change to 450 mg EGCG t.i.d. + 200 mg vitamin C + 20 mg piperine each time. (E) December 2008: start continuous ambulatory peritoneal dialysis (CAPD); EGCG plasma levels decreased significantly, at the same time high EGCG levels were found in CAPD lavage fluid. (F) July 2009: transurethral prostatectomy with further decrease EGCG levels. (G) Dosage correction to 900 mg EGCG t.i.d. + 200 mg vitamin C + 20 mg piperine. (H) November 2009: second transurethral prostatectomy and concurrent peritonitis. Further dosage correction with 450 mg EGCG t.i.d. + 200 mg vitamin C + 10 mg piperine. (I) April 2011: final dosage optimizing with 600 mg EGCG t.i.d. + 200 mg vitamin C + 1000 mg omega-3 fatty acids from salmon each time. No significant changes in FCL or INR were observed across the timeline. A significant decrease in IVS thickness under EGCG therapy could be documented.

We tested several options over the last years in an effort to enhance EGCG levels in plasma [6,18]. Scrupulous observation of the personal experiences and continuous queries in the published data were necessary to reach the current knowledge on optimizing EGCG bioavailability. We were able to reach the highest ever level of EGCG in plasma with the combination of EGCG (purity > 94%) without caffeine taken after an overnight fasting period together with 200 mg ascorbic acid and 1000 mg omega-3 fatty acids from salmon [6] (Figure 3). The following aspects should be taken in account for enhancing bioavailability of EGCG.

#### 3.2.1. Humidity and Temperature

Since EGCG degradation is enhanced by relative humidity and temperature [22], capsules should be stored in cool and dry places, as well as hermetically closed recipients to avoid enhanced oxidation with air contact.

#### 3.2.2. Intake with Empty Stomach

One oral dose in the morning after an overnight fasting period [17,18] at least 30 min before breakfast, and a second dose in the afternoon with fasting period of at least 4 h, at least 30 min before dinner, seem to be necessary to reach high plasma levels of EGCG [6,18].

#### 3.2.3. Hard Waters

Since elevated concentration of calcium, magnesium and other metals in water would affect EGCG absorption [11], it is advisable to soften drinking hard waters with commercial available methods.

#### 3.2.4. Vitamin C

Ascorbic acid alone may improve EGCG bioavailability [6,18] by preventing oxidation, the addition of sucrose may even accentuate this effect by enhancing its absorption in the digestive tract [26].

#### 3.2.5. Fish Oil

Omega-3 polyunsaturated fatty acids may enhance not only oral bioavailability of EGCG but may also improve its efficacy as shown in previous studies [27–29].

#### 3.2.6. Piperine

This black pepper alkaloid could serve as a potential dietary modulator of the bioavailability of EGCG by inhibiting its glucuronidation in the small intestines, as well as inhibit gastric emptying and gastrointestinal transit, which may result in increased absorption [6,18,30].

Further efforts in improving EGCG bioavailability with promising approaches are being undertaken, such as the encapsulation of EGCG in chitosan nanoparticles, enhancing intestinal absorption through polyphenol stabilization [31], or the design and semisynthesis O-acyl derivatives of EGCG [32], or the solid-phase synthesis of EGCG derivatives [33], or even considering another application way, for instance the transdermal delivery of EGCG [34].

## 4. Side Effects of EGCG

Providing EGCG is a pharmakon (ancient Greek word meaning at the same time *remedy* and *poison*), side effects should also be expected. There is an important lack of clinical studies referring to dose levels for different diseases, daily administration frequency and side effects in long term therapy. A daily dose of 800 mg caffeine free EGCG for 4 weeks was shown to be safe and well tolerated in healthy human subjects [25]. However, inherent to different effects of EGCG in several levels, and due to possible contamination with herbicides and/or pesticides, some degree of side effects can be expected:

### 4.1. Anxiolytic Activity

EGCG produces some behavioral effects corresponding to a benzodiazepine-like profile, inducing anxiolytic activity which could result from an interaction with γ-aminobutyric acid (GABA_A_) receptors [35–37]. In addition to an interaction with benzodiazepin receptors, EGCG may inhibit spontaneous excitatory synaptic transmission independently of GABA receptor activation. This inhibition could be due to a direct antagonism of glutamate receptors. Alternatively, EGCG could decrease spontaneous synaptic transmission by attenuating neuronal firing by hyperpolarization as shown in medial vestibular neurons [37].

### 4.2. Hypoglycemic Activity

EGCG suppresses hepatic gluconeogenesis at concentrations that are not toxic to hepatocytes and are reachable by ingestion of green tea or pure EGCG through 5′-AMP-activated protein kinase mediated by the Ca^2+^/calmodulin-dependent protein kinase, both dependent on production of reactive oxygen species [38]. Furthermore, on one hand EGCG mimics insulin action on the transcription factor Forkhead box protein O1 (FOXO1a) and elicits in this way cellular responses in the presence and absence of insulin [39] and on the other hand, EGCG inhibits β-2-aminobicyclo-(2.2.1)-heptane-2-carboxylic acid (BCH) stimulated insulin secretion by pancreatic β-cells mediated by glutamate dehydrogenase [40]. These effects acting together with anxiolytic activity could explain some cases of dizziness referred to by patients taking high oral EGCG doses.

### 4.3. Hypochromic Anemia

Phenolic compounds such as EGCG are potent inhibitors of iron absorption in a dose-dependent fashion depending on the content of total polyphenols, interfering with its assimilation by the complex formation of the gastro-intestinal lumen [41–43].

### 4.4. Liver and Kidney Failure

EGCG and other phenolic compounds may be hepatotoxic at higher doses [44,45]. Moreover, hepatotoxicity related to the consumption of high doses of tea-based dietary supplements (10–29 mg/kg/day) has been reported [46], but the involvement of potentially hepatotoxic agents such as endosulfan, an inexpensive organochlorine pesticide that builds up in the environment extensively used in some green tea plantations [47], was not conclusively ruled out. Chronic exposure to low doses of endosulfan in an additive manner may be hepatotoxic, as well as nephrotoxic [48], but clinical studies in humans are lacking, due to evident ethical considerations.

## 5. Conclusions

The introduction of great therapeutic agents into modern clinical practice [49], as well as the scientific development of new therapy options in medical history started frequently on the basis of naturally available products. Even though green tea health benefits have been known for thousands of years, basic research findings on its main active component, EGCG, have proven to be extremely promising in numerous fields in the last decades. Furthermore, current knowledge force us to go on from just drinking daily large amounts of green tea, or taking over-the-counter green tea extract products, to prescribing medically manufactured pure EGCG, especially for parenteral application, or in combination with enhancers of its bioavailability.

The numerous health benefits of EGCG as a prophylactic, but also as a therapeutic, agent acting through different pathways are well documented in the literature. Conflicting epidemiological inferences and discrepancies between *in vitro* and *in vivo* studies may be due to its erratic bioavailability. Aspects concerning these facts, but also relating to dose levels, administration frequency and potential side effects remain to be addressed in future clinical trials.
